# [Corrigendum] Identification of TYMS as a promoting factor of retroperitoneal liposarcoma progression: Bioinformatics analysis and biological evidence

**DOI:** 10.3892/or.2025.8968

**Published:** 2025-08-06

**Authors:** Sha Zhang, Liang Yan, Can Cui, Zhen Wang, Jianhui Wu, Min Zhao, Bin Dong, Xiaoya Guan, Xiuyun Tian, Chunyi Hao

Oncol Rep 44: 565–576, 2020; DOI: 10.3892/or.2020.7635

Subsequently to the publication of the above paper, the authors contacted the Editorial Office to explain that, for the Transwell migration and invasion assay experiments shown in Fig. 4 on p. 573, the data panel for the ‘94T778 / Lenti-shTYMS-1’ experiment shown in Fig. 4A was selected incorrectly. The authors have re-examined their original data, and realize how this error occurred. The revised (and corrected) version of Fig. 4 shown on the next page. The authors sincerely apologize for the error introduced during the preparation of this figure, although they confirm that this did not grossly affect either the results or the conclusions reported in this study. They also thank the Editor of *Oncology Reports* for granting them the opportunity to publish a Corrigendum, and apologize to the readership for any inconvenience caused.

## Figures and Tables

**Figure 4.**
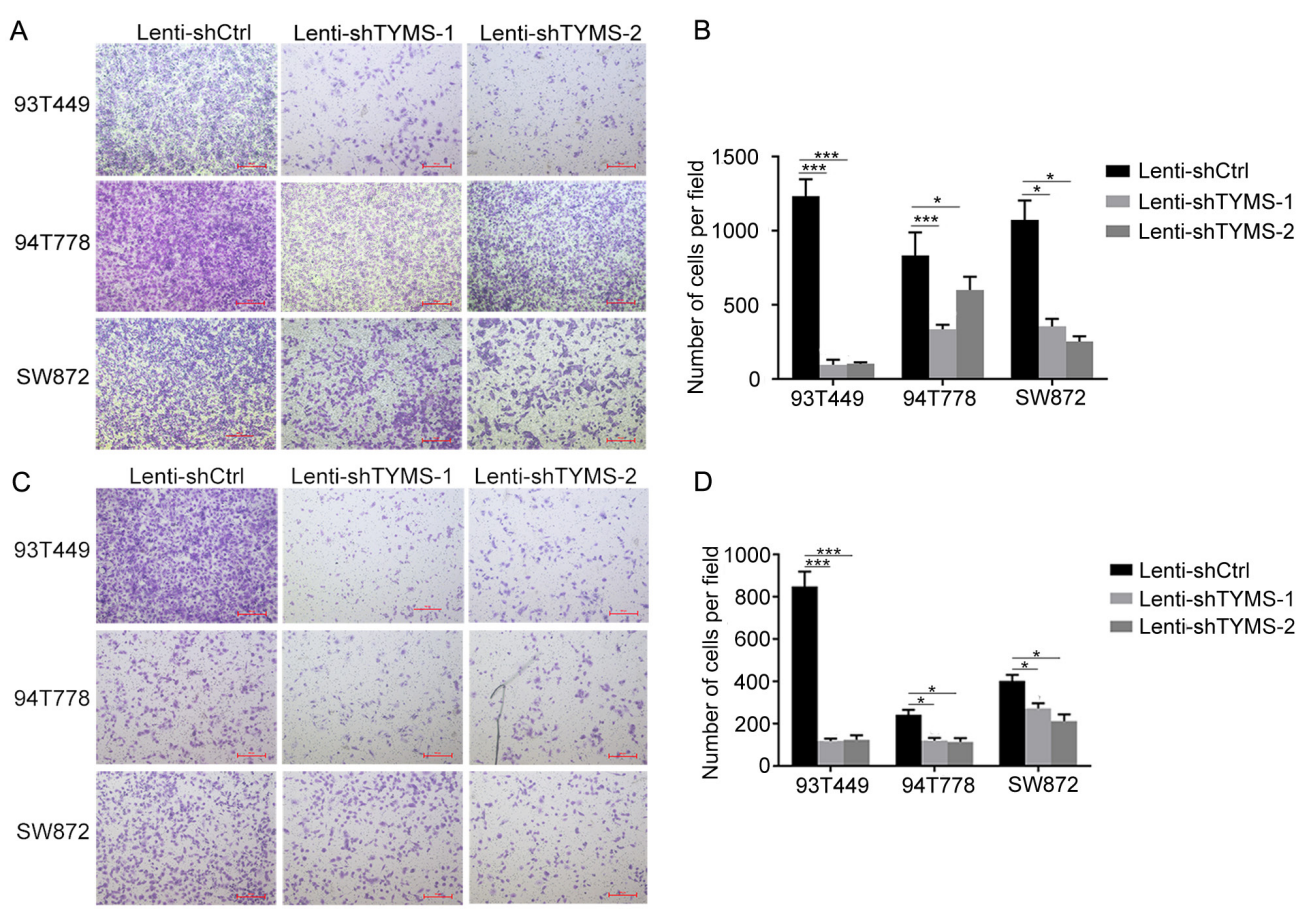
Knockdown of TYMS decreases migration and invasion of RLPS cells. Transwell migration and invasion assays were used to assess the migratory and invasive capacity of RLPS cells. The cells in five randomly selected fields were counted and statistically analyzed. (A and B) The number of migrated cells per field was fewer in Lenti-shTYMS cells compared with Lenti-shCtrl cells of 93T449, 94T778 and SW872 cells. (C and D) The number of invaded cells per field was fewer in Lenti-shTYMS cells compared with Lenti-shCtrl cells of 93T449, 94T778 and SW872 cells. *P<0.05. ***P<0.001. Scale bar, 100 µm. TYMS, thymidylate synthase; RLPS, retroperitoneal soft tissue sarcoma; sh, short hairpin.

